# Facile Microwave-assisted Synthesis Manganese Doped Zinc Sulfide Nanoparticles

**DOI:** 10.1038/s41598-018-34268-z

**Published:** 2018-10-30

**Authors:** David Magalhães Sousa, Luís Cerqueira Alves, Ana Marques, Guilherme Gaspar, João Carlos Lima, Isabel Ferreira

**Affiliations:** 1 CENIMAT/I3N, Departamento de Ciência dos Materiais Universidade Nova de Lisboa Quinta da Torre, Campus Universitário, 2829-516 Caparica, Portugal; 2 Centro de Ciências e Tecnologias Nucleares Instituto Superior Técnico Estrada Nacional 10 (km 139,7), 2695-066 Bobadela, LRS Portugal; 3 LAQV-REQUIMTE, Departamento de Química, CQF B Universidade Nova de Lisboa Quinta da Torre, Campus Universitário, 2829-516 Caparica, Portugal

## Abstract

Undoped and manganese doped zinc sulfide nanoparticles were produced by a fast, one-step and two-component microwave-assisted synthesis method. The solid phase retains around 78% of the initial Mn concentration, as revealed by Particle Induced X-ray Emission analysis. X-ray diffraction patterns confirmed zinc blende structure and in the transmission electron microscopy images, nanoparticles with triangular prism and cube shapes were observed, respectively with an average particle size around 7 nm and 13 nm. Dried powders of zinc sulfide nanoparticles, doped with 0.1 mol% and 0.7 mol% of Mn ions, show highest brilliance of luminescence under UV light. Increasing dopant levels resulted in a diminishing emission that vanishes above 4% of dopant concentration. The synthesis of ZnS was monitored and two main events were detected, one at 145 °C corresponding to the sol-gel phase formation and another after ~3 min at 300 °C where the precipitation of the zinc sulfide nanoparticles occurs.

## Introduction

Zinc sulfide (ZnS) nanoparticles (NPs) with excellent photoluminescence properties are promising for optoelectronic devices, catalysis and sensors^1^ applications. The chemical stability^2^ and synthesis from salts of low toxicity and environmental impact such as zinc acetate^3^, lead ZnS NPs to be ideal also for medical and green technological applications. Being a favorable host for other ions^4^, it is possible to fine tune the bandgap and the luminescence intensity through doping. Zinc sulfide has been doped with transition metal impurities^5^, such as Mn or Cu, in order to achieve new emission properties. Copper doping leads to an increased blue and orange emission^6^, manganese doping selectively enables orange emission and suppresses the blue emission^7^, while white emission is obtained when the particles are co-doped with both Cu and Mn ions^8^.

Given the outstanding luminescence and tailorability of zinc sulfide nanoparticles, it is imperative to develop simple, robust and fast synthesis methods that yield nanoparticles with a well defined geometry and emission properties. Microwave-assisted synthesis has proven to be an expedite route to obtain nanoparticles with these characteristics, since it allows a fast and homogeneous heating during the reaction^9^. ZnS NPs, doped^10^ or undoped^11^, have been previously synthesized in a microwave reactor. The reported methods involve single precursors^12^, which yield good quality nanoparticles, but are either expensive or hard to synthesize. Other synthesis involve hazardous, *e.g*. hydrazine^12^, or toxic, *e.g*. thioacetamide^13^ reagents. Usually, a constant microwave power is applied for a predefined period, until the desired synthesis temperature is reached. There is no control of temperature and pressure over time. In this work, the microwave reactor apparatus used for the synthesis automatically controls the temperature. Taking advantage of the image recording capabilities of the microwave reactor, an in-house developed script was used to analyze the captured images of the reaction vessel, yielding a color profile of the reaction over time. Human observation of a synthesis progression is limited specially when the reactants and the product have the same color^14^. In a color profile, where the red, green and blue pixel color values of the images are plotted against the synthesis time, any slight change is more easily detected. This allowed us to access the intermediate products by repeating the synthesis at the temperature where the color change occurred. This procedure is a simpler and cheaper alternative to the more sophisticated *in situ* spectroscopy^14^.

A different approach to the previously reported microwave-assisted ZnS nanoparticle synthesis is proposed and the results reported. 1-Dodecanethiol, a long chain alkylthiol with low toxicity and mild reductant properties, has been previously used as a sulfide source but it was dissolved in 1-octadecene, together with zinc acetylacetonate^15^. In this work, another strategy was attempted, the system was simplified to two components, comprising the non-toxic zinc acetate and 1-dodecanethiol acting as solvent, reductant, sulfur source and stabilizer, simultaneously.

## Results and Discussion

**Figure 1:**
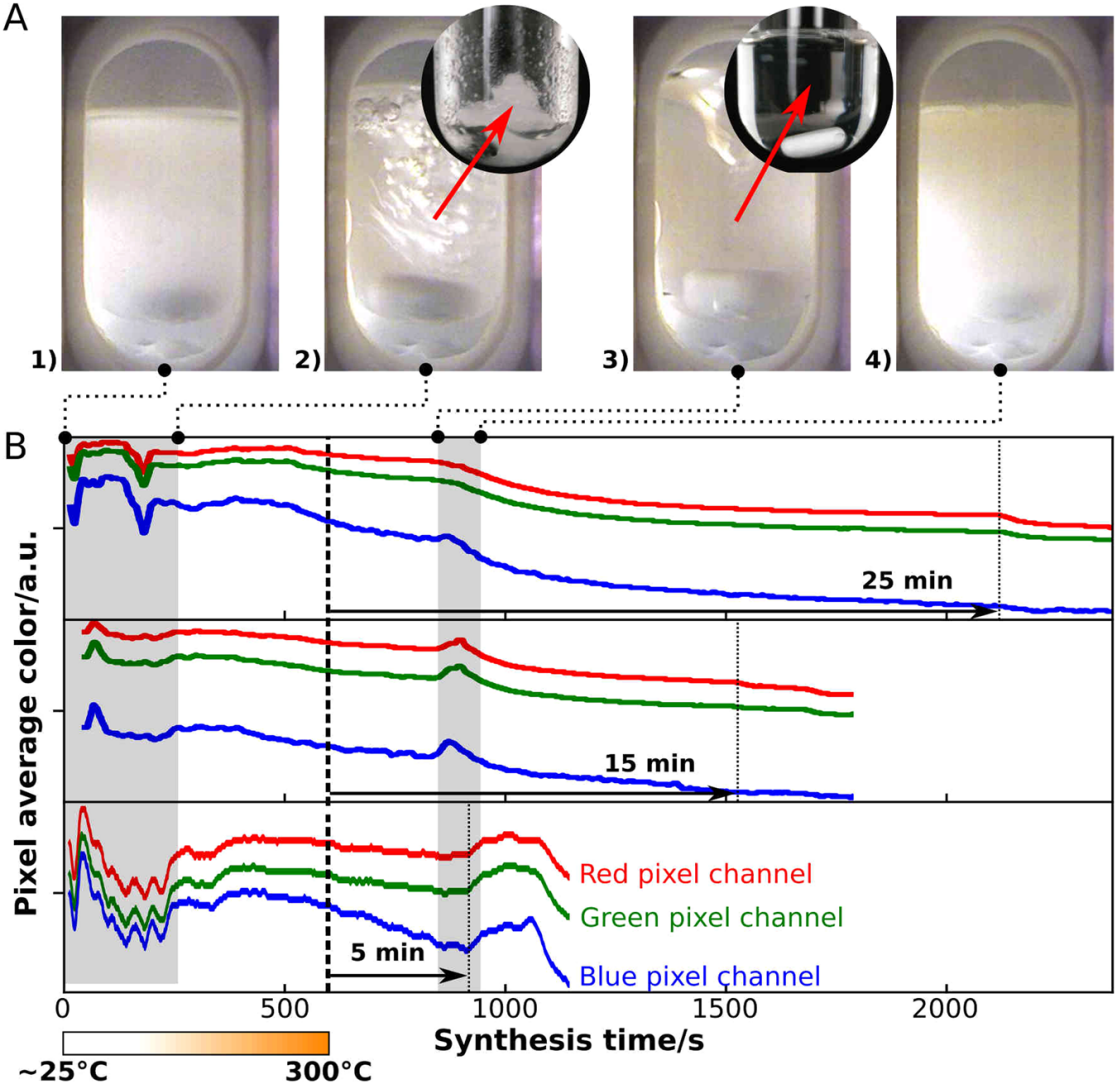
(**A**) Pictures of the microwave assisted zinc sulfide nanoparticle synthesis at different synthesis times, from left to right: (1) heterogeneous mixture of the precursor and solvent, (2) gel formation, (3) gel dissolution and (4) precipitation of ZnS. Each picture corresponds to the beginning or the end of the darkened areas. (**B**) Color profiles of the synthesis at 300 °C for 5 min, 15 min and 25 min, representing the red, green and blue average pixel values taken from the recorded images, as a function of synthesis time. The bar below the plot indicates the synthesis temperature ramp.

The zinc sulfide nanoparticles were obtained by microwave-assisted heating of a reactant mixture of zinc acetate (219.5 mg) in 1-dodecanethiol (5 g) at 300 °C for 25 min. Doping with manganese was achieved by adding manganese acetate tetrahydrate to the previous mixture, in molar concentrations ranging from 0.05% to 10%. An approximate average yield of 84 ± 16 mg was obtained, after washing the synthesized zinc sulfide nanoparticles, corresponding to a 86% molar yield. The camera integrated in the microwave reactor enabled capturing images during the reaction evolution, as shown in the depicted examples in Fig. 1A. The images were later processed by a script which extracts the average red, green and blue pixel color values of the whole image and conjugates it with the synthesis temperature as a function of synthesis time. The resulting plot, Fig. 1B, enables a fine observation of the reaction, since any visible event displays a change in the red, green and blue average pixel color values at a precise time frame.

The mixture observed in the first images of each synthesis are usually heterogeneous, since the reaction vessel is heating up and the zinc salt is not dissolved in the solvent, as shown in Fig. 1A1). This produces images that vary significantly each second, until the salt is dissolved. Even the gel that is formed yields pictures that vary from synthesis to synthesis. Therefore, in the first 250 s the color profiles are dissimilar but merely due to the heterogeneous property of the mixture. After complete dissolution of the semi-solid structures, the color profile stabilizes. As ZnS nanoparticle synthesis starts, two main events were observed. The first is the fast formation of a gel like structure, Fig. 1A2), with no visible particles, as observed in the transmission electron microscopy (TEM) images, Figure S1A of the Supplementary Information. Zinc sulfide does not form at temperatures lower than 150 °C, as 1-dodecanethiol only starts to release the sulfide ion above that temperature^16^. The gel dispersed after 3 min of synthesis time, at around 145 °C, and completely dissolved at 215 °C after 6 min, resulting in a transparent suspension, Figure 1A3). The TEM image, Figure S1B, of the precipitate from the latter suspension, Figure 1A4), shows very small and sporadic triangular particles, indicating the beginning of the nucleation process. The second event was associated to the slight color variation, consistently occurring after 13 min to 15 min from the beginning of the synthesis, where the nanoparticle begun to grow.

Color profiles can be used to detect reactions, changes of physical state and check for synthesis reproducibility. The synthesis carried out in this work only differ in synthesis time after reaching the 300 °C target (5 min, 15 min or 25 min). Several changes are observed during the synthesis occurring in well defined time frames, following the temperature profile shown in Figure S2 of the Supplementary Information. As reported by other authors^14^, this type of digital recording and processing greatly enhances the overall control over synthesis.

**Figure 2:**
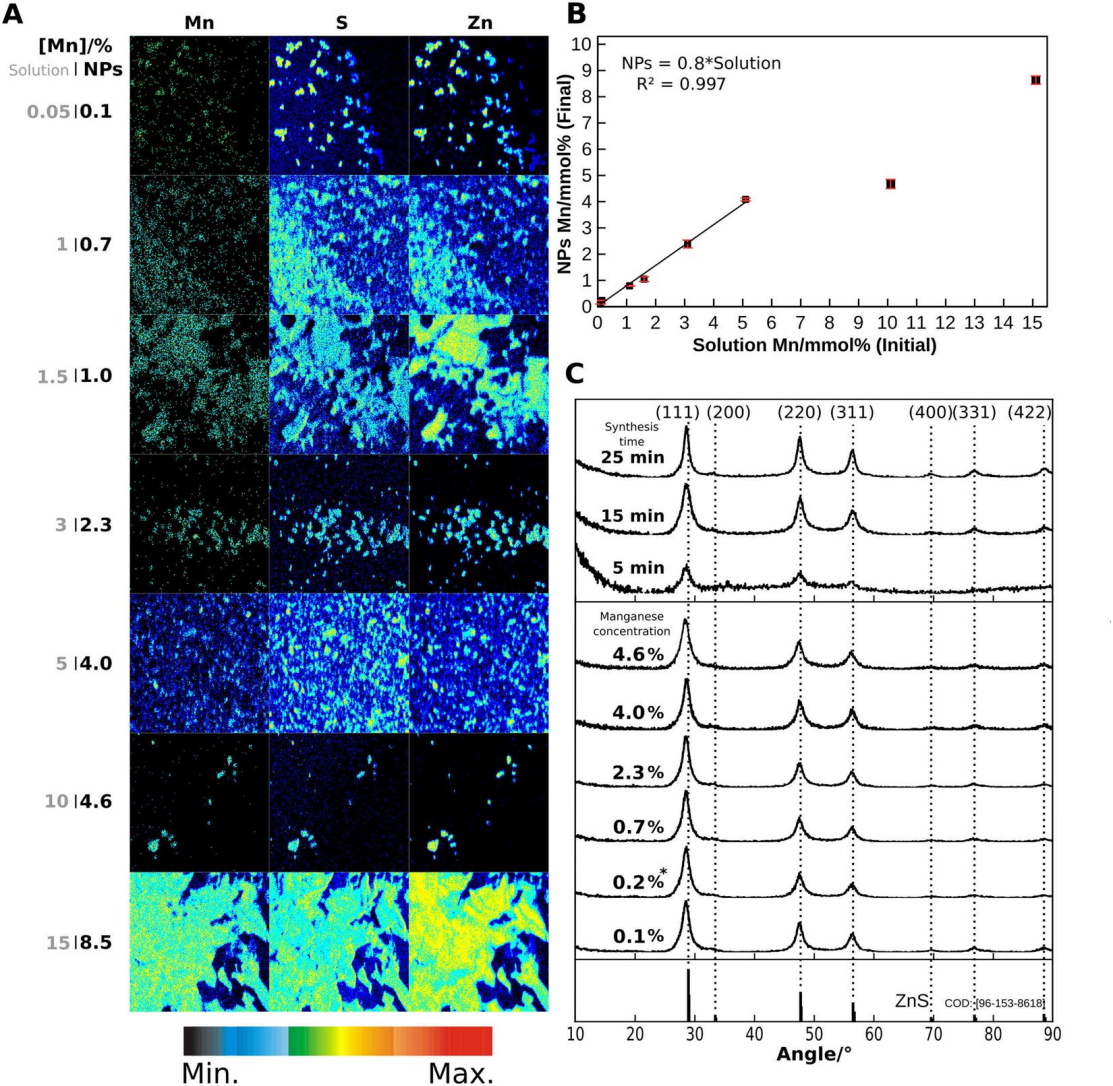
(**A**) Images of powder samples PIXE relative intensity, in a 1320 × 1320 µm^2^ area, of manganese, sulfur and zinc. The initial (gray) and final (black) manganese percentages are shown beside each sample. The bottom color bar represents the relative PIXE intensity. (**B**) Plot of the previous percentages, including estimated error bars, and linear correlation from 0.05% to 5% initial concentration. (**C**) X-ray diffraction patterns of undoped and doped zinc sulfide nanoparticles synthesized for 5 min, 15 min and 25 min at 300 °C and different concentrations of manganese, for 25 min at 300 °C. The vertical dashed lines denote the corresponding crystal planes and the reference pattern of ZnS sphalerite is included in the bottom. The asterisk denotes an extrapolated concentration value from PIXE results, corresponding to 0.2% initial manganese concentration.

The obtained powders were dispersed in chloroform and drop cast onto a polyimide substrate, to measured zinc, sulfur and manganese concentrations by PIXE, in the conditions referred in Materials and Methods section and on the Supplementary Information Figure S3 and text. Fig. 2A shows the induced X-ray emission relative intensity maps acquired on powder lumps deposited by drop-cast, over an area of 1320 × 1320 µm^2^. The pixel intensity, represented by the color code bar, corresponds to the elemental detected X-ray yield and is proportional to the elemental concentration, as shown in the Supplementary Information. Images corresponding to manganese, sulfur and zinc show a great elemental spatial correlation and, therefore, manganese is mostly near zinc on a microscopic scale. At the nanoscopic scale PIXE cannot evidently prove that Mn is somehow bonded or incorporated inside the nanoparticles but allows determining the correlation between molar percentage in solution and the obtained washed powder, as shown in Fig. 2B. It has a direct proportionality of 0.8, up to 5% of manganese concentration in the solution, meaning that about 20% of the Mn precursor is not incorporated into the nanoparticles. As will be shown later in this article, an emission effect is observed in this range up to 5%, above which it greatly diminishes. Hence, a linear fit has been made only within this limit of interest demonstrating the usefulness of PIXE analysis on the prediction of the final Mn concentration for any initial chosen amount of Mn. The higher initial concentrations of Mn of 10% and 15% were included to check if the loss of Mn would follow the previous trend. As a matter of fact, around 50% of the initial Mn concentration is lost.

**Figure 3:**
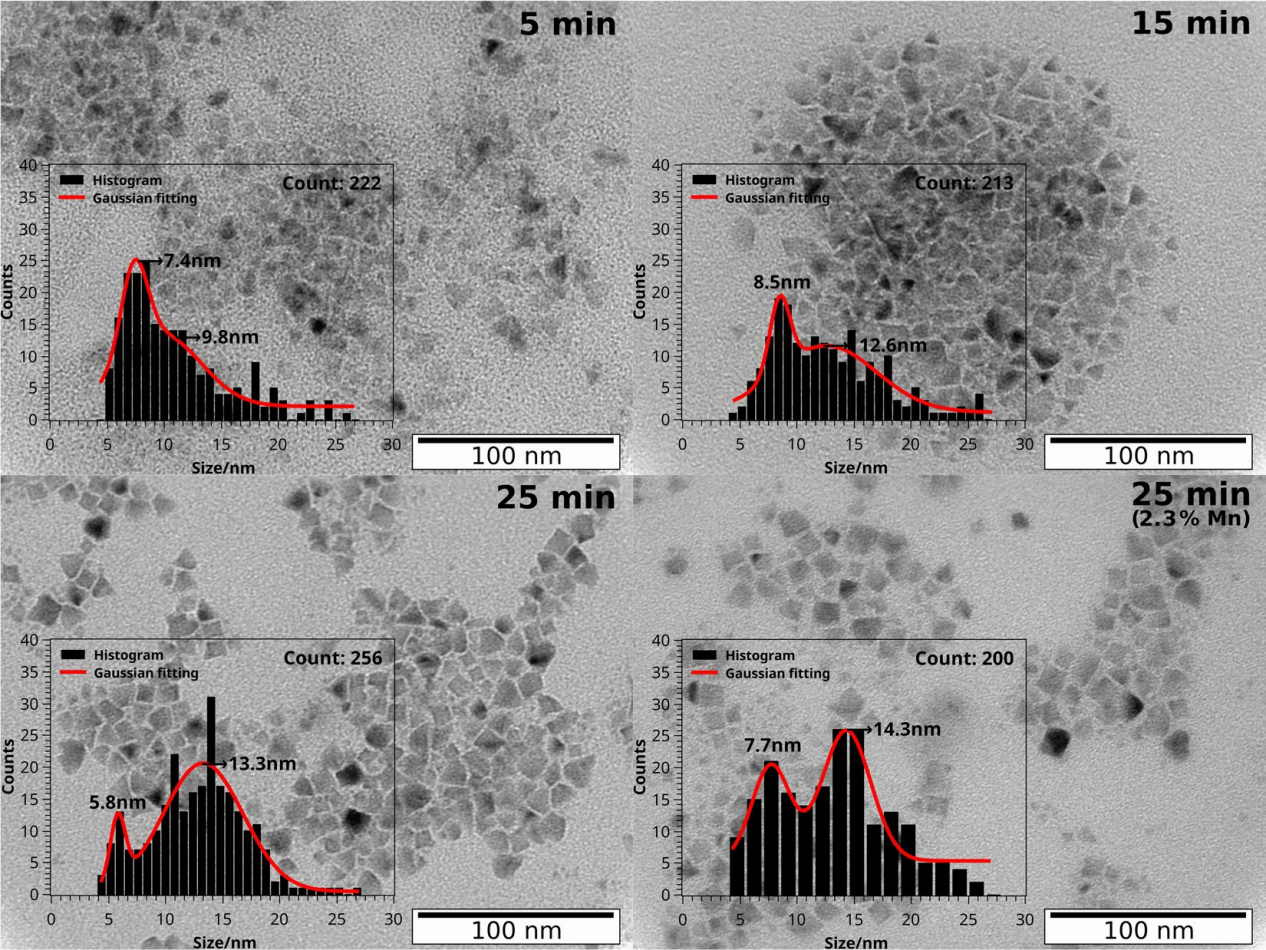
TEM images with nanoparticle size histograms of zinc sulfide nanoparticles synthesized for 5 min, 15 min and 25 min at 300 °C and zinc sulfide nanoparticles doped with 2.3% of manganese, synthesized for 25 min at 300 °C.

Synthesis time influences on the crystalline structure of the obtained nanoparticles as shown in Fig. 2C. However, dopant concentration does not seem to affect the structure according to the resulting pattern. The X-ray diffraction (XRD) patterns show the main Bragg’s reflection peaks at 2θ of 28.68°, 33.11°, 47.70°, 56.47°, 69.80°, 76.98°, and 88.67° that correspond to the planes (111), (200), (220), (311), (400), (331) and (422). These planes are characteristic of the zinc blende (cubic) phase of ZnS, also know as Sphalerite, which is the expected to be formed at 300 °C^17^. The obtained 2θ values are in accordance with standards JCPD N° 79–43^18^ and PDF N° 05–0566)^19^. No significant deviation from the reference patterns is seen for the undoped particles synthesized for 25 min at 300 °C, relative to the 2θ value of the three most intense peaks, corresponding to the planes (111), (220) and (311). Although no phase transformation occurs when the particles are doped with manganese, the three main peaks have a constant −0.19° ± 0.02° shift. The same shift is observed for the undoped particles synthesized for 5 min at 300 °C. The estimated crystallite size for the undoped material is 6.1 nm, 4.5 nm and 8.6 nm for the 5 min, 15 min and 25 min synthesis at 300 °C, respectively. For doped nanoparticles crystallite sizes ranges from 4.8 to 6.7 nm. Therefore, we can conclude that crystallite size of the produced ZnS nanoparticles are in the range of 4 to 8 nm and have a minor influence of synthesis time and doping. The influence of synthesis time (5 min, 15 min and 25 min at 300 °C) and Mn doping at 2.3% on the shape of ZnS NPs, can be observed on the transmission electron microscope images of Fig. 3. The ZnS NPs present triangular and cubic shapes. The average size distribution estimated from the TEM images is 5–9 nm for the triangular particles and 12–14 nm for the cubic particles, as depicted in the figure.

The triangle shape of the nanoparticles is owned to 1-dodecanethiol, as obtained in similar synthesis^15,20,21^. On the other hand a co-mixture of oleylamine with 1-dodecanethiol yields spherical particles^16,20^. 1-Dodecanethiol slowly decomposes to yield sulfur at high temperatures^16^, which can contribute to a lower size distribution of the nanoparticles at shorter synthesis times. Indeed, the size distribution is centered at around 5–7 nm when synthesis time is 5 min, it is still present as synthesis time increases but a new population of larger cubic shaped nanoparticles emerges, becoming the major population when the synthesis time is 25 min at 300 °C. The size distribution of the triangular particles agrees with the crystallite size range determined by XRD, however, the appearance of cubic particles suggests they are formed by aggregation of triangular particles as their size almost duplicates and XRD crystalline size is not affected by synthesis time.

The nanoparticles shape and average size are not significantly influenced by doping according TEM images and respective size distribution of undoped, 0.7% (Figure S4B Supplementary Information S4 B) and 2.3% (Fig. 3) doped ZnS NPs.

**Figure 4:**
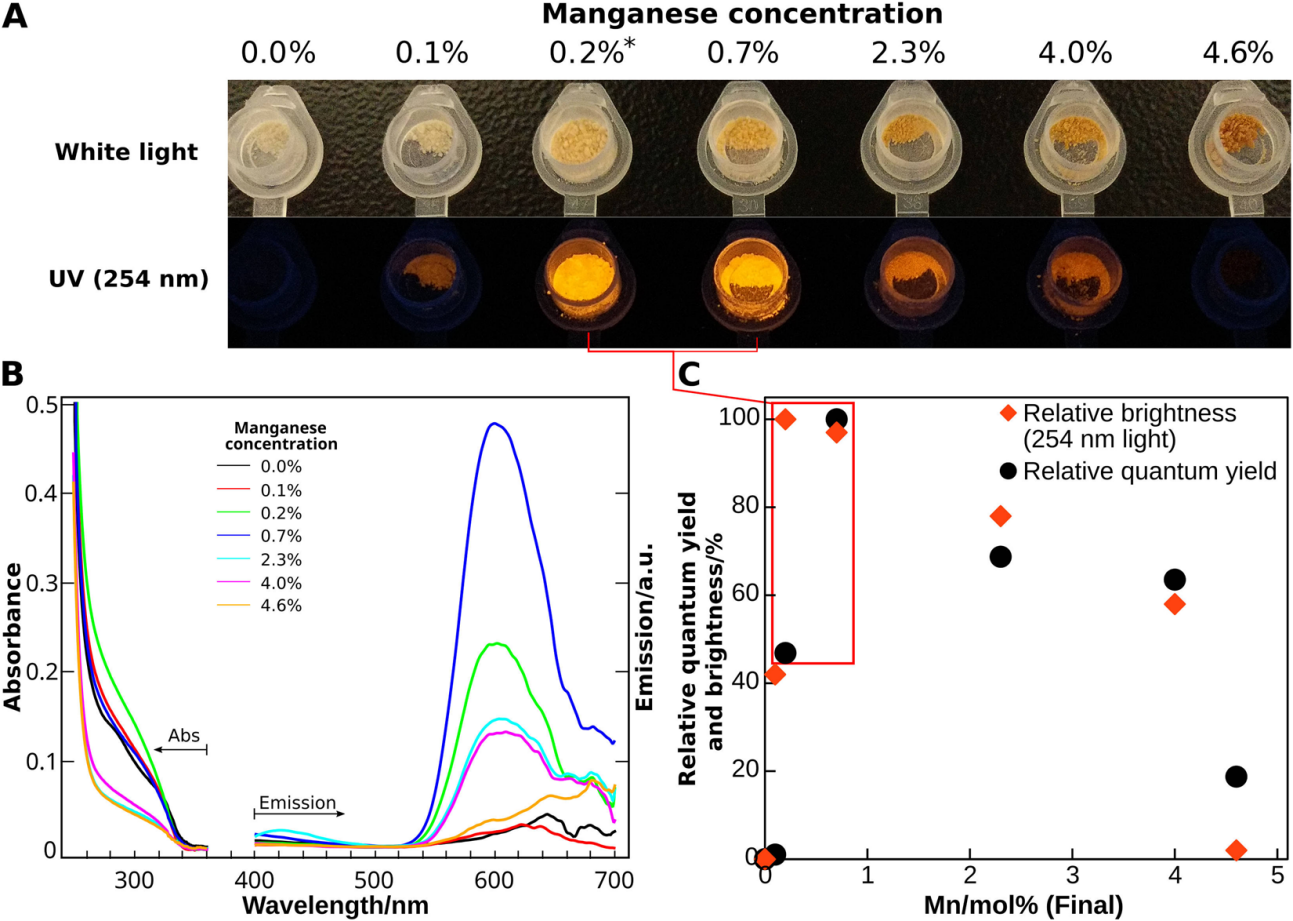
(**A**) Pictures of the doped and undoped zinc sulfide nanoparticle dried dusts, irradiated under white light and 254 nm UV lights. (**B**) Absorption and emission spectra of the zinc sulfide nanoparticles undoped and doped with manganese in concentrations ranging from 0.1% to 4.6% suspended in chloroform, with excitation wavelength of 300 nm and a 360 nm cut-off filter. (**C**) Final concentration of manganese in moles versus the relative quantum yield and pixel intensity of yellow/orange. The asterisk denotes an extrapolated concentration value from PIXE results, corresponding to 0.2% initial manganese concentration.

A first analysis on how Mn doping influences the luminescence of ZnS NPs was obtained by observing the dried powders under ambient light and irradiated by a 254 nm wavelength UV lamp, as shown in Fig. 4A. The absorption and emission spectra of undoped and doped zinc sulfide nanoparticles are shown in Fig. 4B, while the pixel intensity obtained from the pictures and the quantum yield is correlated in Fig. 4C. The absorption spectra, found in Figure S5A, were corrected using Castanho *et al*.^22^ algorithm to remove the turbidity effect, scattering of light from the ethanolic suspension, and reveal the absorption features of the nanoparticles. Bandgaps were determined via Tauc’s plots, as found in Figure S5.

Undoped zinc sulfide nanoparticles clearly do not emit any visible light, as can be seen in Fig. 4A. With the introduction of a small amount of dopant a faint emission and brilliance are obtained. When the concentration of Mn reaches 0.1% the highest brilliance is attained. The maximum quantum yield is reached at 0.7%, below the 3% reported by others^23^, and the powder emits an intense light. As the concentration of dopant increases beyond 0.7%, the nanoparticles emission brightness decreases and the powder progressively turns dark brown. Darkening of the powder is predominantly observed for the last two concentrations, which is a consequence of the higher initial manganese concentration. As the powder becomes darker, both the relative quantum yield and the powder brightness decreases, in agreement with previous results^23^.

The bandgap of bulk zinc sulfide (cubic phase) is 3.68 eV, according to Fang *et al*.^24^ but for nanoparticles, it can go as low as 3.40 eV due to quantum confinement, defects, local bonds, intrinsic surface states and interfaces^18^. The estimated bandgap for the synthesized nanoparticles is between 3.51 eV and 3.60 eV, as shown in Figure S5. No considerable dependence of the bandgap value on dopant concentration is observed, as opposed to the emission intensity. An emission band centered at 600 nm, typically of manganese doped zinc sulfide^7,10^, explains the orange emission observed when the particles are irradiated with UV light. Energy transfer from the zinc sulfide to the d-orbital of the manganese impurities occurs via two paths: from the zinc sulfide conduction band and from the defect states. After the non-radiative energy transfer step, the decay of manganese excited states originates the observed orange emission^7^. This is compatible with the reported suppression of the blue emission of ZnS when the particles are doped, since the available energy is redirected to the manganese ions. However, a dominant blue emission was not observed in undoped ZnS nanoparticles, as reported previously by Sapra *et al*.^7^. A slightly higher blue emission intensity is observed from the doped nanoparticles, especially the ones doped with 2.3% manganese, compared to the undoped ones. Emission centered at 420 nm is usually attributed to surface defects of the nanoparticles^25^ while the tail up to 500 nm is related to sulfur vacancies^26^. Inconsistency is observed in this emission region, as seen in Figure S5B, between the manganese concentration and the emission intensity. This suggests that defects are originated on the synthesis process, albeit with low impact on the blue emission intensity, which has no apparent correlation with manganese impurity concentration.

## Materials and Methods

Zinc acetate dihydrate (reagent grade), 1-dodecanethiol (≥98%) and spectroscopic grade chloroform were purchased from Sigma-Aldrich. Ethanol absolute anhydrous (ACS-Reag., Ph.Eur.-Reag. USP) used for washing the nanoparticles was purchased from CARLO ERBA Reagents. Manganese acetate tetrahydrate (Mn 22%) was obtained from Alpha Aeser. The nanoparticle synthesis was performed in an Anton Paar Microwave Synthesis Reactor Monowave 400 and pictures of the synthesis were recorded using the reactor’s integrated camera. The script built to process the captured pictures was written in Python programming language (version 3.5.5) and used Python Imaging Library (version 1.1.7), also known as PIL.

Zinc sulfide nanoparticles were synthesized by addition of 1 mmol of zinc acetate dihydrate to 5 g of 1-dodecanethiol, in an appropriate 15 mL microwave-friendly glass vessel, capped with a silicon rubber septum. A small magnet was also added to stir the mixture. The mixture was degassed for 15 min under a constant flow of industrial grade nitrogen gas, supplied by Air Liquide and under vigorous agitation, while the vessel was uncapped. After closing the vessel and inserting it in the microwave reactor, the mixture was subject to heating to 300 °C in 10 min. Once the temperature was reached, it was kept for 5 min, 15 min or 25 min. Cool-down took an average of 4 min to reach 50 °C. Washing was performed four times by adding anhydrous ethanol, circa 20 mL, followed by centrifugation at 9000 RPM for 10 min. The particles were then dried under vacuum for 2 h. The obtained particle dusts were stored under ambient conditions in a closed vessel. The experiment was repeated in order to introduce manganese acetate dihydrate, as the doping material. The initial concentrations of dopant [0.05, 0.2, 1, 1.5, 3, 5, 10, 15]% were relative to 1 mmol of zinc acetate dihydrate. For example, for the 1% initial Mn concentration case, 0.01 mmol of manganese acetate dihydrate was added to 1 mmol of zinc acetate dihydrate and 5 g 1-dodecanethiol.

Particle-induced X-ray emission data was obtained through an Oxform Microbeams scanning nuclear microprobe (OM150 type) using a 2 MeV proton beam generated by a 2.5 MV Van de Graaff accelerator, to determine the final manganese concentration after the synthesis and washing procedures. Samples were prepared by suspending ~1 mg of each nanoparticle powder in chloroform, followed by dropping the suspension onto a 2 cm × 2 cm polyimide substrate.

Nanoparticles zinc blende (cubic; Sphalerite) structure was confirmed by X-ray diffraction in a X’Pert PRO MPD, with a Cu Kalpha-1 source at 1.540598 Å wavelength. The Lorentz function *y* = *y0* + 2 * *A*/π * *B*/(4*(*x*-*xc*)^2^ + *B*^2^) was fitted to the most intense peaks, located at around 28.6°, 47.7° and 56.4°, respectively corresponding to the planes (111), (220) and (311). In this function, *y0* corresponds to the peak baseline, A is the integrated area of the peak and, most importantly, *B* is the full width at half maximum and *xc* is the 2θ value of the peak center. The *B* and *xc* parameters obtained from the fittings done to the selected peaks were used to determine the crystallite size by the Williamson-Hall method. By plotting B*cosθ as a function of sinθ, the resulting intercept from the linear fit is K*λ/L, which directly yields the crystallite size *L*, considering a shape factor *K* of 0.94 and a XRD source wavelength *λ* of 1.540598 Å. Note that the instrumental effect on peak broadening was neglected.

Transmission electron microscopy images were obtained from a Hitachi H-8100 II with a 200 kV electron beam, after depositing an ethanolic suspension of the nanoparticles on a Formvar and carbon coated 200mesh copper grids.

Absorption spectra were obtained from a Perkin Elmer Lambda 35 UV-Vis Spectrophotometer and emission spectra from a Perkin Elmer LS45 Luminescence Spectrometer, from a suspension of ~1 mg of powder in 100 mL of chloroform. Absolute quantum yields, not shown here, were determined using 7-diethylamino-4-methylcoumarin in ethanol (96%) as a reference and for each dopant concentration ~1 mg of powder was suspended in 100 mL of chloroform. Quantum yield determination was performed without degassing. A cutoff filter at 360 nm was used to cut harmonic artifacts below 620 nm and a neutral density filter of 1 Abs unit to prevent the saturation of the light sensor. The relative quantum yields were obtained by the normalization of the absolute values.

## Conclusion

Zinc sulfide nanoparticles doped with manganese were obtained by a simple, two-component and fast microwave-assisted synthesis, with a 86% molar yield. Almost 78% of the initial manganese remained in the solid phase after the synthesis procedure, as estimated by PIXE analysis. Two size distributions of around 7 nm and 14 nm were observed in the TEM images, respectively corresponding to triangular and cubic shapes, typical of the Sphalerite phase which was identified by XRD for undoped and Mn doped ZnS NPs. The estimated crystallite size is in the range of 4–8 nm independently of the Mn concentration and synthesis time. Irradiating the doped nanoparticles with UV light resulted in an orange emission, centered at 600 nm. The brightest orange emission and relative quantum yield was achieved when ZnS was doped with Mn at concentrations of 0.1% and 0.7%. Overall, the almost absent blue light emission reflects the low concentration of defects in the nanoparticles.

A script was developed to analyze the images recorded by the camera inside the reactor, which allows correlating the temperature variation and the average red, green and blue pixel color values as a function of synthesis time. This allowed the detection of different synthesis events, namely the dissolution of the reagent and formation of a gel, followed by the dissolution of the gel and the precipitation of zinc sulfide nanoparticles.

## Electronic supplementary material

supplementary information
