# Supplementary material for: Facile Microwave-assisted Synthesis Manganese Doped Zinc Sulfide Nanoparticles
Source: Sci Rep. 2018 Oct 30;8:15992. doi: 10.1038/s41598-018-34268-z (PMC6207788; doi:10.1038/s41598-018-34268-z)
Supplement: Supplementary file 1 — supplementary information [file 41598_2018_34268_MOESM1_ESM.pdf]

Facile Microwave-assisted Synthesis Manganese Doped Zinc Sulfide Nanoparticles (supplementary information)

David Magalhães Sousa*[a], Luís Cerqueira Alves[b], Ana Marques[a], Guilherme Gaspar[a], João Carlos Lima[c], Isabel Ferreira[a]

[a] David Magalhães Sousa, Dr. Ana Marques, Dr. Guilherme Gaspar, Prof. Isabel Ferreira
CENIMAT/I3N, Departamento de Ciência dos Materiais
Universidade Nova de Lisboa
Quinta da Torre, Campus Universitário, 2829-516 Caparica, Portugal
*Corresponding author e-mail: dma.sousa@campus.fct.unl.pt

[b] Prof. Luís Cerqueira Alves
Centro de Ciências e Tecnologias Nucleares
Instituto Superior Técnico
Estrada Nacional 10 (km 139,7), 2695-066 Bobadela LRS, Portugal

[c] Prof. João Carlos Lima
LAQV-REQUIMTE, Departamento de Química, CQF B
Universidade Nova de Lisboa
Quinta da Torre, Campus Universitário, 2829-516 Caparica, Portugal

Supplementary information to the article "**Facile Microwave-assisted Synthesis Manganese Doped Zinc Sulfide Nanoparticles**" is presented in this document, pertaining details about the synthesis method and Particle Induced X-ray Emission (PIXE). Additional transmission electron microscopy (TEM) images of zinc sulfide (ZnS) nanoparticle synthesis, as well as results of the synthesis using solely manganese acetate tetrahydrate are also shown. Finally, the absorption and emission spectra of the nanoparticles, coupled with the Tauc's plots for bandgaps calculations, are presented.

Table of contents - Figures

Figure S1 - A - TEM image of the gel formed in the synthesis using zinc acetate dihydrate and 1-dodecanethiol, stopped when the temperature reached 100 ºC. B - TEM image of the suspension formed in the synthesis using zinc acetate dihydrate and 1-dodecanethiol, stopped when the temperature reached 300 ºC....2
Figure S2 - Temperature profile over time of the zinc sulfide nanoparticle synthesis done with an initial ramp to 300 ºC in 10 min and retaining the temperature for 5 min, 15 min or 25 min, and the profile of the synthesis including manganese ions....2
Figure S3 - Schematic overview of the micro Particle Induced X-ray Emission (PIXE) technique, illustrating the (a) radiation detection system arrangement and the proton beam scanning pattern, (b) the total X-ray spectrum recorded during the whole sample scanning with the selected spectrum regions of interest to evaluate pixel contribution and perform 2D elemental distribution mapping, (c) the elemental concentration maps - where the pixels intensity correspond to the detected X-ray yield, and (d) the general X-ray yield equation for thick samples (i.e. thicker than a few µm) clearly evidencing the direct proportionality between the yield and the elemental concentration calculated by the program GUPIX....3
Figure S4 - A - TEM image of the powder obtained from a synthesis under the same conditions as undoped zinc sulfide nanoparticles, using solely manganese acetate tetrahydrate as a precursor, and corresponding XRD pattern. B - TEM image with nanoparticle size histogram of zinc sulfide nanoparticles doped with 0.7% manganese ions....5
Figure S5 - A – As measured absorbance spectra of the zinc sulfide nanoparticles, undoped and doped with various concentrations of manganese. B - Emission of the zinc sulfide nanoparticles, undoped and doped with various concentrations of manganese, between 400 nm and 500nm, with excitation at 360 nm. The asterisk denotes an extrapolated concentration value from PIXE results, corresponding to 0.2% initial manganese concentration....6
Figure S6 - Tauc plots of the zinc sulfide nanoparticles, undoped and doped with various concentrations of manganese. Linear regression was taken from the interval between 3.51 eV and 3.60 eV to determine the bandgap value by extrapolation (αhν)1/2 = 0. The asterisk denotes an extrapolated concentration value from PIXE results, corresponding to 0.2% initial manganese concentration....7

Synthesis details

The following figures comprise TEM images of the various ZnS nanoparticle synthesis phases, at different temperatures.

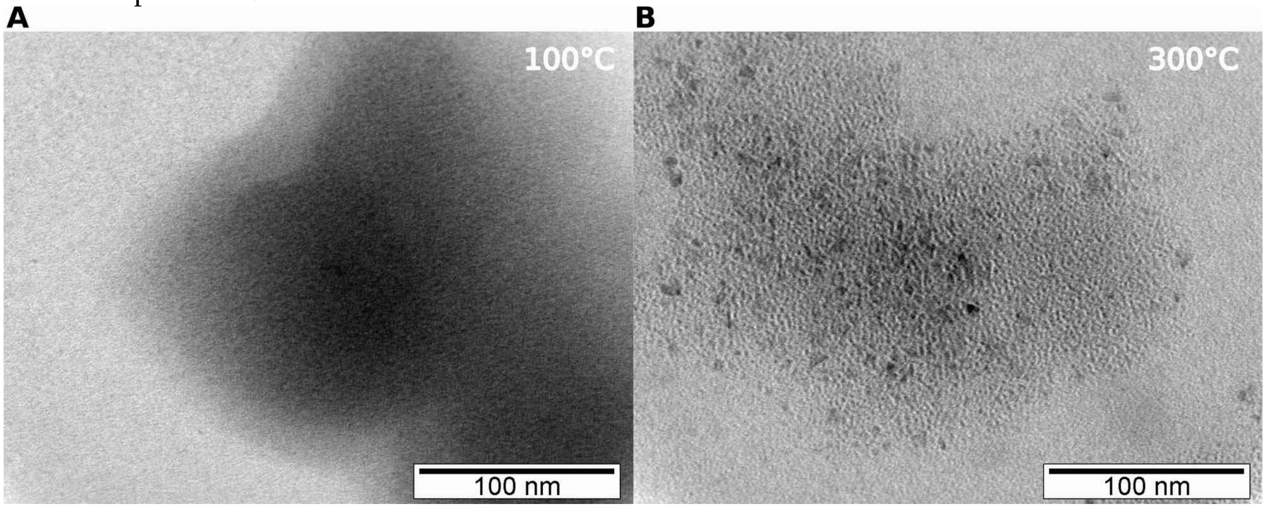

*Figure S1 - **A** - TEM image of the gel formed in the synthesis using zinc acetate dihydrate and 1-dodecanethiol, stopped when the temperature reached 100 ºC. **B** - TEM image of the suspension formed in the synthesis using zinc acetate dihydrate and 1-dodecanethiol, stopped when the temperature reached 300 ºC.*

The TEM image of the first synthesis stage where a gel is formed is shown in Figure S1 A, where no nanoparticles are seen, as expected. Figure S1 B corresponds to the TEM image of a synthesis stopped at 300 ºC, where the first nanoparticles are formed.

The setup used on the synthesis of nanoparticles was an Anton Paar Microwave Synthesis Reactor Monowave 400. The system automatically modulates the power supplied to the microwave antenna, such that the temperature of the reaction vessel, which monitored by an infrared sensor, is brought to the per-programmed value in a given time. The power supplied to the antenna is therefore variable but the temperature profile over time, from synthesis to synthesis, is kept constant (Figure S2). The average power supplied on the 10 min ramp to 300 ºC was 48±2 W, with a spike of 166±18 W on the first 15 seconds of synthesis. After the 300 ºC temperature was reached, a stable 38±2 W of power is supplied to the microwave antenna to maintain the temperature.

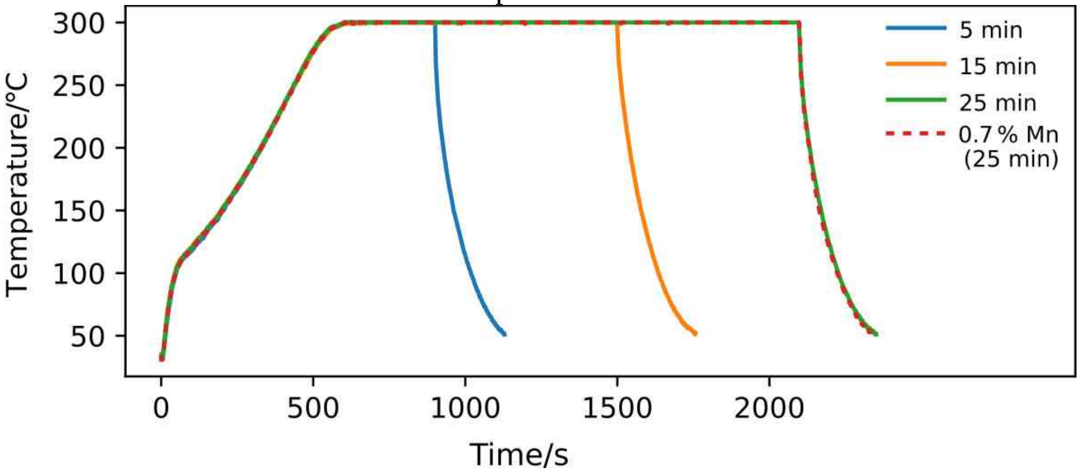

Figure S2 - Temperature profile over time of the zinc sulfide nanoparticle synthesis done with an initial ramp to 300 ºC in 10 min and retaining the temperature for 5 min, 15 min or 25 min, and the profile of the synthesis including manganese ions.

Particle-induced X-ray Emission

The PIXE analysis was performed with the aim of determining the concentration of Mn after its incorporation during the synthesis of Mn-doped ZnS powders. The initial concentration of Mn was varied in each synthesis, *i.e*. from 0.5% to 15%, and the correspondent PIXE analysis was performed. PIXE spectra were obtained under vacuum (10^{-6} mbar) at the microprobe beam line of the CTN/IST 2.5 MV Van der Graaff Accelerator, using a 2 MeV proton beam at normal incidence with a cross section of 3×3 µm^2. The Si(Li) detector of 145 eV resolution was placed at 135° to the beam direction and was operated with a 8 µm thick Be window and a 50 µm thick Mylar filter, to prevent protons from entering the detector active area. The surface elemental distribution maps were obtained as explained in Figure S3 and they show a clear spatial correlation between S, Zn and Mn. The beam was scanned over samples areas of 1320×1320 µm^2 by means of a digital scanning system from Oxford Microbeams, and the data collection run on an event by event basis under the control of the OM-DAQ software. In the selected regions of interest, PIXE spectra evaluation and quantification was done with the GUPIX code. The results include the elemental concentration obtained for each sample as listed in Figure 2 in the main article.

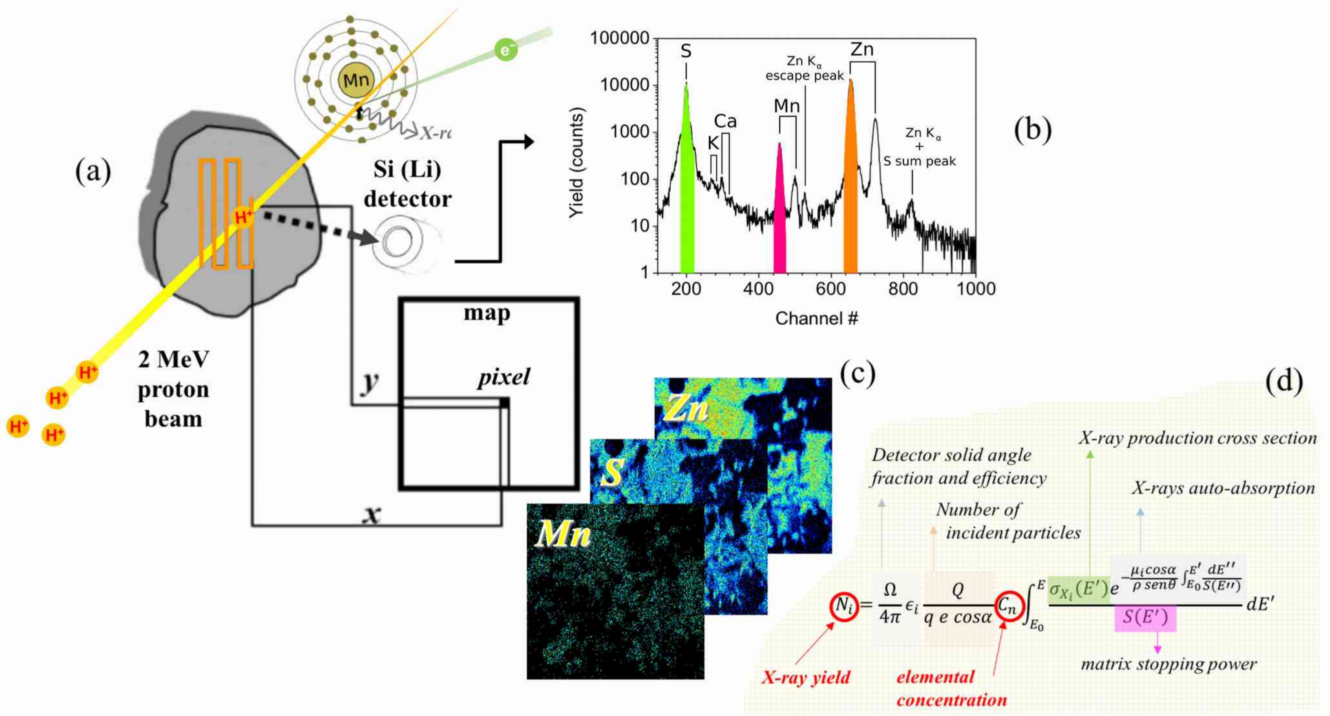

Figure S3 - Schematic overview of the micro Particle Induced X-ray Emission (PIXE) technique, illustrating the (a) radiation detection system arrangement and the proton beam scanning pattern, (b) the total X-ray spectrum recorded during the whole sample scanning with the selected spectrum regions of interest to evaluate pixel contribution and perform 2D elemental distribution mapping, (c) the elemental concentration maps - where the pixels intensity correspond to the detected X-ray yield, and (d) the general X-ray yield equation for thick samples (i.e. thicker than a few µm) clearly evidencing the direct proportionality between the yield and the elemental concentration calculated by the program GUPIX.

For further details about the PIXE technique, theory and data processing the reader is now invited to read reference [1]. Nevertheless, generally speaking, the number of detected elemental X-ray (spectrum peak area) is a function of the sample elemental concentration as expressed in the inset of Figure S3, and calculation is performed using a fundamental parameters algorithm. Depending on the sample thickness, the integral variable E (proton beam energy after crossing sample material) can generally

take values from 0 (zero – thick sample approximation) up to E_0 (initial proton beam energy – no beam energy loss and the integral can be simplified to the thin sample approximation). The calculations can be performed either for a nano-sized particle film (thin target approximation) or up to the range of the used particle beam (thick target) that for most materials represents thicknesses up to ~30 µm. For samples with intermediate thickness, proton energy exit (E) can be converted into sample thickness. For the samples analyzed in this work, a strategy for determining the concentration of the Mn/Zn ratio and the error majorant of this determination was followed. First find the thin sample Mn/Zn ratio and then use an intermediate thick sample with sample thickness iteratively altered until S and Zn are in a stoichiometric relation (1:1). The two values found should be the minimum and maximum acceptable Mn/Zn concentration ratios.

1. Johansson, S.A.E., Campbell, J.L., & Malmqvist, K.G. *Particle-Induced X-ray Emission Spectrometry (PIXE). Chemical Analysis, a series of monographs on analytical chemistry and its applications* (ed. Winefordnen, J.D.) (John Wiley & Sons, Ltd, 1995).

Manganese doping

A synthesis was performed under the same conditions, as described in the main article, using 1 mmol of manganese acetate tetrahydrate in 1-dodecanethiol and without zinc acetate dihydrate. In Figure S4 A lies a TEM image and XRD pattern of the obtained powder.

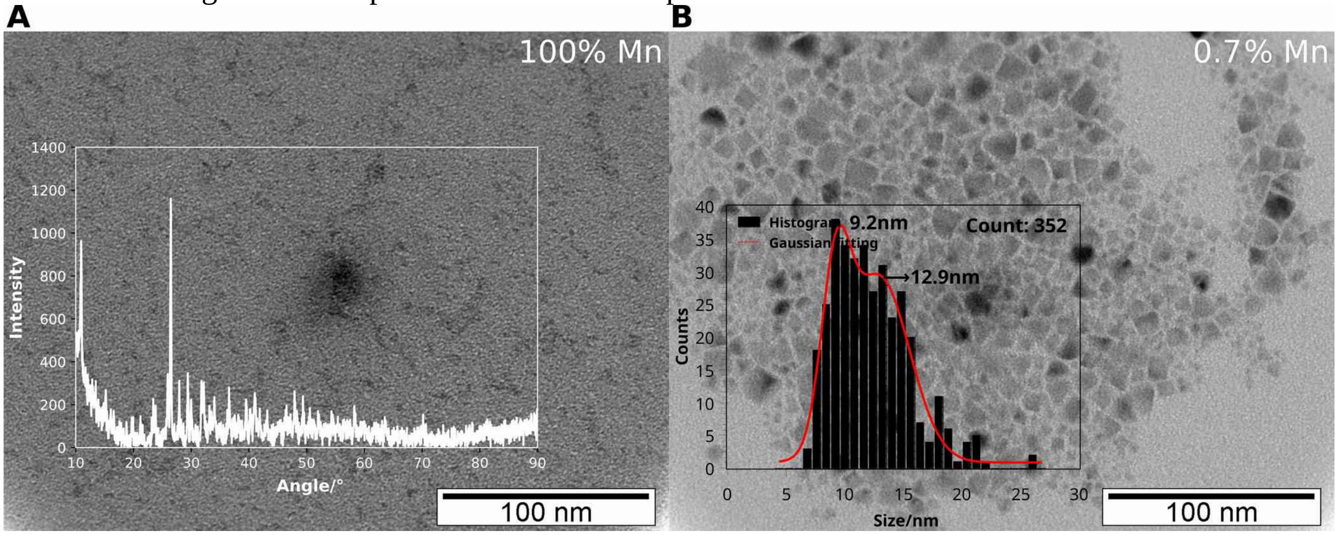

*Figure S4 - **A** - TEM image of the powder obtained from a synthesis under the same conditions as undoped zinc sulfide nanoparticles, using solely manganese acetate tetrahydrate as a precursor, and corresponding XRD pattern. **B** - TEM image with nanoparticle size histogram of zinc sulfide nanoparticles doped with 0.7% manganese ions.*

Figure S4 B corresponds to a TEM image of ZnS nanoparticles doped with an initial concentration of 0.7% of manganese ions, with the purpose of showing that no visible changes occurred, in respect to undoped particles.

Optical properties

This section is dedicated to complement the spectroscopic information presented in the main article body.

Figure S5 corresponds to the as measured spectra of the doped and undoped ZnS nanoparticles and the corresponding emission in the range of 400-500 nm.

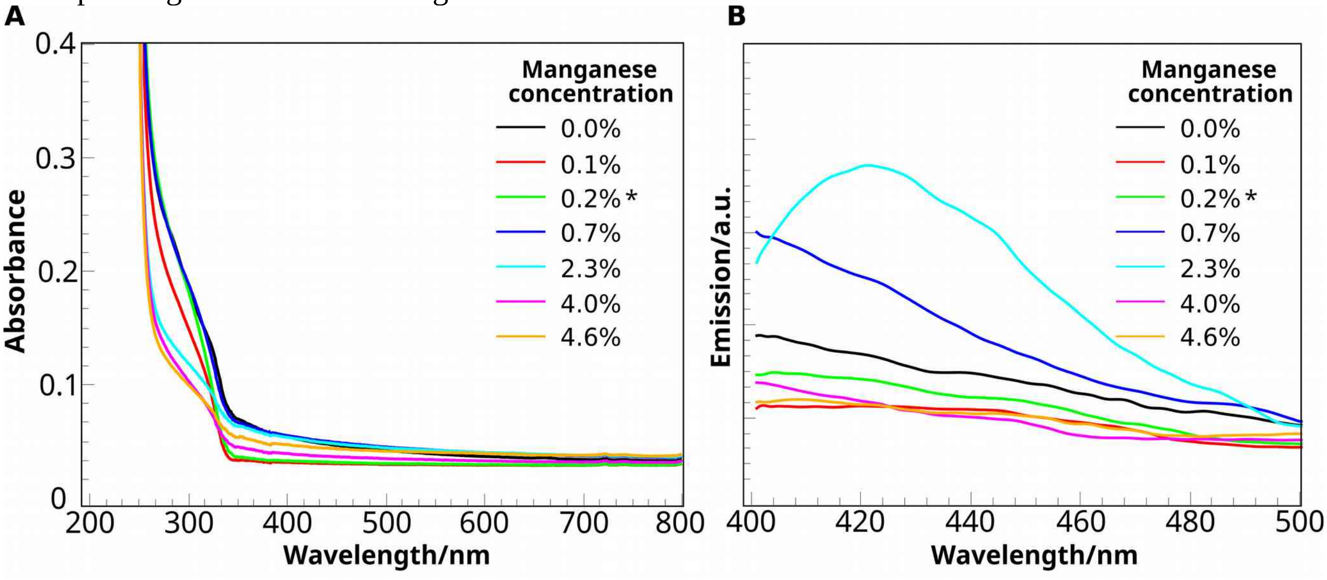

Figure S5 - ***A*** *– As measured absorbance spectra of the zinc sulfide nanoparticles, undoped and doped with various concentrations of manganese.* ***B*** *- Emission of the zinc sulfide nanoparticles, undoped and doped with various concentrations of manganese, between 400 nm and 500nm, with excitation at 360 nm. The asterisk denotes an extrapolated concentration value from PIXE results, corresponding to 0.2% initial manganese concentration.*

The correction of the spectra consisted in subtracting a polynomial, fitted to the absorption regions corresponding to scattered light > 400 nm (Figure S5 A). Emission spectra from the synthesized doped and undoped ZnS nanoparticles, as shown in Figure S5 B, demonstrates that there is no correlation between the initial manganese doping concentration and the blue emission, i.e. at around 420 nm.

The Tauc plots shown in Figure S6, were used to obtain the synthesized materials bandgaps. The linear segment of each spectrum was determined from the first derivative of the corresponding data. The regions where the derivative is zero were denoted as the start and end of the linear segment of the curves. From the linear region of $(\alpha h\nu)^{1/2}$ as a function of photon energy (hν) the bandgap was directly obtained at the point where the fit crossed the hν axis.

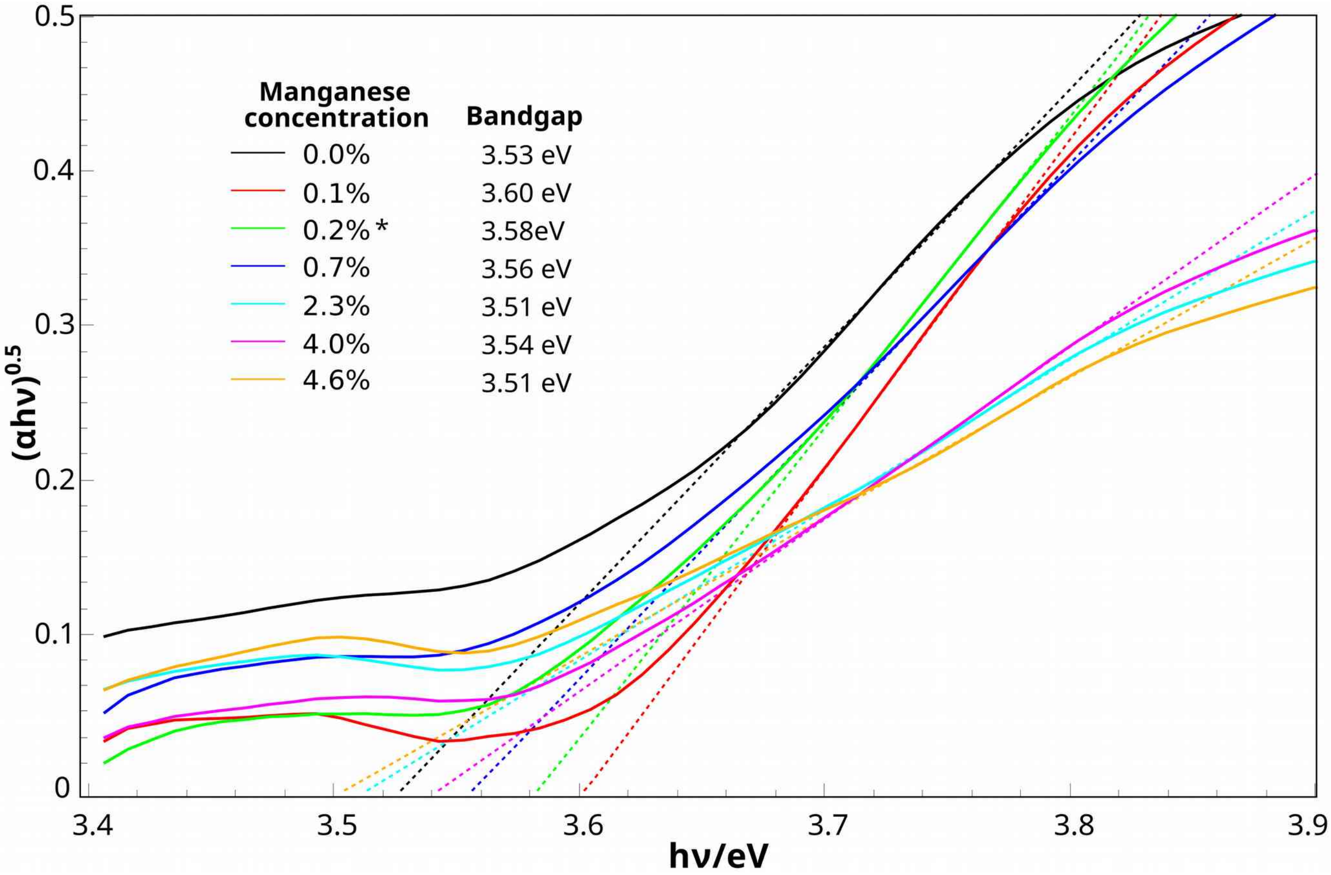

Figure S6 - Tauc plots of the zinc sulfide nanoparticles, undoped and doped with various concentrations of manganese. Linear regression was taken from the interval between 3.51 eV and 3.60 eV to determine the bandgap value by extrapolation $(\alpha h\nu)^{1/2} = 0$. The asterisk denotes an extrapolated concentration value from PIXE results, corresponding to 0.2% initial manganese concentration.
